# James Alexander

**Published:** 1905-06

**Authors:** 


### JAMES ALEXANDER.

The death of James Alexander, of Paignton, took place after a short illness on April 2nd, in his fifty-eighth year.

He graduated M.B., M.Ch. Dublin in 1869, and M.D. in 1878. His death has removed from amongst us one of no ordinary character, for he seems to have possessed in uncommon degree the power of gaining the affections as well as the confidence of all those with whom he came in contact. Somewhere about 1871 he accepted the appointment of Surgeon to the Union Mining Company of Newfoundland, and Newfoundland is not a country that lends itself to the romance, either of medicine or anything else; nevertheless, our deceased friend so won his way into people's hearts there, that on the termination of his engagement with the Company, one of the proprietors, Mr. Smith McKay, entrusted him with the custody and education of his son, whom he brought home with him when he settled at Paignton. Both of the mining proprietors, viz. Mr. C. Fox Bennett and Mr. Smith McKay, have pre-deceased him, but both would have borne willing testimony to his worth as a medical man, and as guide, counsellor, and friend.

As to his life at Paignton, Dr. Alexander was connected with most of the social and philanthropic work of the place, including the School Board, of which he was Chairman; and the esteem in which he was held there is sufficiently shown by the very large gathering that attended at his funeral. It is a happy termination to the career of any medical man when it can be truthfully inscribed on his headstone: "He was *loved* as well as *trusted*."

